# Comparison of genotyping using pooled DNA samples (allelotyping) and individual genotyping using the affymetrix genome-wide human SNP array 6.0

**DOI:** 10.1186/1471-2164-14-506

**Published:** 2013-07-26

**Authors:** Alexander Teumer, Florian D Ernst, Anja Wiechert, Katharina Uhr, Matthias Nauck, Astrid Petersmann, Henry Völzke, Uwe Völker, Georg Homuth

**Affiliations:** 1 Interfaculty Institute for Genetics and Functional Genomics, University Medicine Greifswald, 17487 Greifswald, Germany; 2 Department of Medical Oncology, Erasmus MC, 3008AE, Rotterdam, The Netherlands; 3 Institute of Clinical Chemistry and Laboratory Medicine, University Medicine Greifswald, 17475 Greifswald, Germany; 4 Institute for Community Medicine, University Medicine Greifswald, 17475 Greifswald, Germany

## Abstract

**Background:**

Genome-wide association studies (GWAS) using array-based genotyping technology are widely used to identify genetic loci associated with complex diseases or other phenotypes. The costs of GWAS projects based on individual genotyping are still comparatively high and increase with the size of study populations. Genotyping using pooled DNA samples, as also being referred as to allelotyping approach, offers an alternative at affordable costs. In the present study, data from 100 DNA samples individually genotyped with the Affymetrix Genome-Wide Human SNP Array 6.0 were used to estimate the error of the pooling approach by comparing the results with those obtained using the same array type but DNA pools each composed of 50 of the same samples. Newly developed and established methods for signal intensity correction were applied. Furthermore, the relative allele intensity signals (RAS) obtained by allelotyping were compared to the corresponding values derived from individual genotyping. Similarly, differences in RAS values between pools were determined and compared.

**Results:**

Regardless of the intensity correction method applied, the pooling-specific error of the pool intensity values was larger for single pools than for the comparison of the intensity values of two pools, which reflects the scenario of a case–control study. Using 50 pooled samples and analyzing 10,000 SNPs with a minor allele frequency of >1% and applying the best correction method for the corresponding type of comparison, the 90% quantile (median) of the pooling-specific absolute error of the RAS values for single sub-pools and the SNP-specific difference in allele frequency comparing two pools was 0.064 (0.026) and 0.056 (0.021), respectively.

**Conclusions:**

Correction of the RAS values reduced the error of the RAS values when analyzing single pool intensities. We developed a new correction method with high accuracy but low computational costs. Correction of RAS, however, only marginally reduced the error of true differences between two sample groups and those obtained by allelotyping. Exclusion of SNPs with a minor allele frequency of ≤1% notably reduced the pooling-specific error. Our findings allow for improving the estimation of the pooling-specific error and may help in designing allelotyping studies using the Affymetrix Genome-Wide Human SNP Array 6.0.

## Background

Genome-wide association studies (GWAS) using array-based genome-wide individual single nucleotide polymorphism (SNP) typing technologies are performed to identify genetic loci associated with complex diseases. The costs of projects based on individual genotyping using microarrays are still high and increase directly with the number of samples to be analyzed. GWAS using pooled DNA samples, the so called allelotyping approach, offers an alternative at significantly lower costs [1].

Several earlier studies have described the identification of new or validation of known phenotype-associated genetic loci by allelotyping using older SNP genotyping array versions [2-4]. Recently, findings from allelotyping using newer generations of arrays that enable genome-wide SNP coverage, such as the Affymetrix Genome-Wide Human SNP Array 6.0, were also published [5,6]. In an allelotyping study, the estimated allele frequencies are subject to various quantitative technical errors related to pooling of the DNA samples. These pooling-specific errors result from DNA quantification and generation of pools, amplification of target sequence, and frequency estimation with the chosen methodology [7]. While several studies evaluated the pooling-specific error of former array systems [8-12], error estimated for the Affymetrix Genome-Wide Human SNP Array 6.0 have not been done so far.

Despite the great advantage of cost reduction, allelotyping has also several drawbacks. Most importantly, allelotyping allows only for extraction of the mean allele frequencies of the pooled DNA samples instead of determining the individual genotypes. In addition, adjusting for covariates is almost impossible after DNA pooling. This limitation includes the use of principal components as covariates in the association analysis to adjust for population stratification as commonly done in GWAS. If researchers wish to use the allelotyping approach they need to be certain that little to no stratification exists, or that it will be adequately controlled by e.g. applying genomic control. Furthermore, the error rate is usually higher in pooled samples compared to individual samples. Notwithstanding these weaknesses, allelotyping represents a well-priced alternative, especially when applied to case–control studies with larger cohort sizes, because in these cases, stronger effects of individual gene variants compared to general-population based cohorts can be expected.

In the present study, we estimated the pooling-related error by comparing the allele frequencies determined from pooled DNA samples with those obtained from individually genotyped samples. In both cases the Affymetrix Genome-Wide Human SNP Array 6.0 was used as genotyping platform. Furthermore, we compared differences in allele frequencies between several DNA pools used for allelotyping. Finally, we evaluated some known and newly developed methods for correcting the pooling-specific error and compared these results to the uncorrected allele frequencies. Our results may help researchers set up an appropriate experimental design for case–control allelotyping studies.

## Results

The study population included two groups consisting of 50 individuals each. The DNA samples of each group were either pooled and analyzed using one array, or individually genotyped resulting in 50 arrays per group. In each case, the Affymetrix Genome-Wide Human SNP Array 6.0 was used. We determined the allele frequencies using relative allele signals (RAS) [10] (see also Methods section). The original RAS value calculations were modified for the Affymetrix Genome-Wide Human SNP Array 6.0. Two different methods were tested for obtaining the A and B allele intensities used for RAS value calculation: the Birdseed2 genotype calling algorithm [13] and the median probe set intensities from the array-specific CEL files (details are given in the Methods section). We estimated the pooling-specific error by comparing the allele frequencies obtained from the allelotyping experiment with the corresponding allele frequencies acquired by individual genotyping. Three different correction methods for the Birdseed2 based RAS values were applied and compared: a) the k-correction method [8,12,14], b) a slightly modified PPC correction algorithm according to [11] and c) our newly developed correction method based on tangent-transformation (tan-correction) using three different parameter values *n* = 0.5,1,2 (see Methods section).

**Figure 1:**
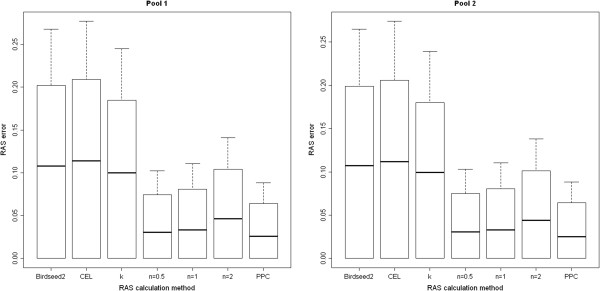
**Pooling-specific error.** The absolute difference (error) of the A allele frequency obtained using pooled DNA samples (y-axis) compared to the expected allele frequency based on individual genotyping using the corresponding RAS calculation method (x-axis). Birdseed2: uncorrected RAS values obtained using the Birdseed2 algorithm; CEL: uncorrected RAS values obtained from CEL files; k: k-corrected RAS value; n: tan-correction using the specified value for *n* as parameter; PPC: PPC corrected RAS values. The horizontal lines represent the median error, the bars show the 90% quantile and the length of the whiskers corresponds to the standard deviation of the error. The plots show the error of DNA-Pool 1 (left) and DNA-Pool 2 (right), estimated using the MAF >1% SNP set.

Comparison of 10,000 uncorrected RAS values calculated from single DNA pools of 50 samples with those derived from individual genotyping calls and a SNP minor allele frequency (MAF) >1% resulted in the 90% quantile (median) of the absolute errors of 0.202 (0.108). Applying the PPC correction method reduced this error to 0.064 (0.026). Our newly developed tan-correction with parameter *n* = 0.5 performed second-best with corresponding absolute errors of 0.074 (0.031). The corresponding error value of the k-correction method amounted to 0.18 (0.1). The comparison of the pooling-specific errors using different correction methods applied on this SNP set is shown in Figure 1.

Using 10,000 RAS values with a MAF >5% increased the allelotyping accuracy slightly, whereas inclusion of all 772,731 SNPs with a genotype call rate of 100% in the analysis strongly increased the overall pooling-specific error. Since the computationally intensive PPC correction algorithm was originally developed for the Affymetrix GeneChip 10 K Mapping array representing approximately 10,000 SNPs, we could not apply this correction method on the complete set of SNPs. For the complete set of SNPs, the tan-correction method with parameter *n* = 0.5 performed best with a 90% quantile (median) error of 0.122 (0.042) compared to 0.25 (0.138) using uncorrected RAS values obtained from the Birdseed2 calling algorithm. The k-correction method slightly increased the accuracy after removing the low-MAF SNPs but did not outperform the accuracy of uncorrected RAS values of the complete SNP set.

The differences of the error distribution between both single DNA-pools varied only marginally. Detailed results are given in Table 1.

**Table 1:** Error of the RAS values of single DNA pools

***Pool 1***	**RAS (Birdseed2)**		**RAS (CEL)**		**RAS (k-corrected)**		**RAS (PPC-corrected)**	
**SNP set**	**Q90 (Median)**	**Mean (SD)**	**Q90 (Median)**	**Mean (SD)**	**Q90 (Median)**	**Mean (SD)**	**Q90 (Median)**	**Mean (SD)**
all SNPs (N = 772731)	0.25 (0.138)	0.141 (0.081)	0.257 (0.144)	0.146 (0.083)	0.267 (0.127)	0.14 (0.09)	NA	NA
10000 SNPs MAF >1%	0.202 (0.108)	0.112 (0.066)	0.209 (0.114)	0.117 (0.068)	0.185 (0.1)	0.103 (0.06)	0.0642 (0.026)	0.031 (0.024)
10000 SNPs MAF >5%	0.192 (0.0981)	0.104 (0.064)	0.199 (0.104)	0.108 (0.065)	0.170 (0.0902)	0.0938 (0.057)	NA	NA
***Pool 1***	**RAS (tan-corrected n = 0.5)**		**RAS (tan-corrected n = 1)**		**RAS (tan-corrected n = 2)**			
**SNP set**	**Q90 (Median)**	**Mean (SD)**	**Q90 (Median)**	**Mean (SD)**	**Q90 (Median)**	**Mean (SD)**		
all SNPs (N = 772731)	0.122 (0.0417)	0.0577 (0.11)	0.13 (0.0438)	0.0628 (0.15)	0.16 (0.0539)	0.11 (1.6)		
10000 SNPs MAF >1%	0.0744 (0.0306)	0.0362 (0.028)	0.0806 (0.0333)	0.0392 (0.03)	0.104 (0.0464)	0.0523 (0.037)		
10000 SNPs MAF >5%	0.0724 (0.0298)	0.0353 (0.027)	0.0783 (0.0325)	0.0382 (0.029)	0.101 (0.046)	0.0515 (0.036)		
***Pool 2***	**RAS (Birdseed2)**		**RAS (CEL)**		**RAS (k-corrected)**		**RAS (PPC-corrected)**	
**SNP set**	**Q90 (Median)**	**Mean (SD)**	**Q90 (Median)**	**Mean (SD)**	**Q90 (Median)**	**Mean (SD)**	**Q90 (Median)**	**Mean (SD)**
all SNPs (N = 772731)	0.252 (0.139)	0.142 (0.082)	0.262 (0.147)	0.149 (0.085)	0.267 (0.128)	0.14 (0.09)	NA	NA
10000 SNPs MAF >1%	0.199 (0.107)	0.110 (0.066)	0.206 (0.112)	0.114 (0.068)	0.18 (0.0993)	0.101 (0.059)	0.0641 (0.0251)	0.0306 (0.024)
10000 SNPs MAF >5%	0.191 (0.0976)	0.103 (0.064)	0.195 (0.101)	0.105 (0.065)	0.165 (0.0893)	0.0914 (0.055)	NA	NA
***Pool 2***	**RAS (tan-corrected n = 0.5)**		**RAS (tan-corrected n = 1)**		**RAS (tan-corrected n = 2)**			
**SNP set**	**Q90 (Median)**	**Mean (SD)**	**Q90 (Median)**	**Mean (SD)**	**Q90 (Median)**	**Mean (SD)**		
all SNPs (N = 772731)	0.123 (0.043)	0.058 (0.061)	0.129 (0.0451)	0.0625 (0.14)	0.157 (0.0548)	0.107 (2.6)		
10000 SNPs MAF >1%	0.075 (0.0304)	0.0363 (0.028)	0.0804 (0.0329)	0.0388 (0.03)	0.101 (0.0442)	0.0506 (0.037)		
10000 SNPs MAF >5%	0.0737 (0.0304)	0.0359 (0.027)	0.0786 (0.0326)	0.0383 (0.029)	0.0993 (0.0442)	0.0499 (0.035)		

Absolute differences (error) between RAS values obtained by allelotyping using DNA-pools and expected RAS values calculated from calls obtained by individual genotyping of the same DNA samples. Q90 represents the 90% quantile, SD the standard deviation of the error.

**Figure 2:**
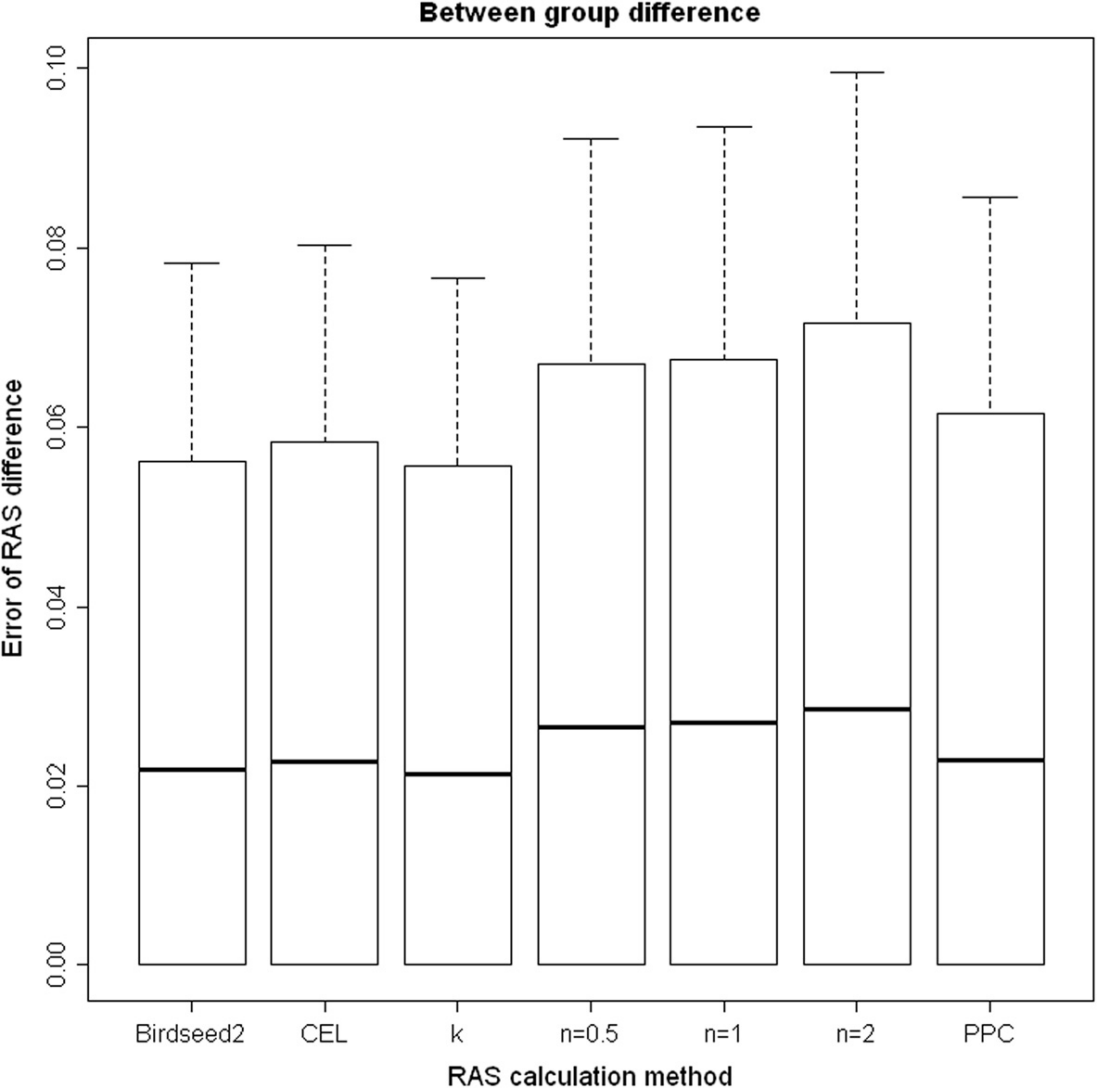
**Error of difference between both DNA-pools.** The absolute difference (error) of the difference between DNA-Pool 1 and DNA-Pool 2 allele frequencies (y-axis) compared to the expected difference of the allele frequency based on individual genotyping using the corresponding RAS calculation method (x-axis). Birdseed2: uncorrected RAS values obtained using the Birdseed2 algorithm; CEL: uncorrected RAS values obtained from CEL files; k: k-corrected RAS value; n: tan-correction using the specified value for *n* as parameter; PPC: PPC corrected RAS values. The horizontal lines represent the median error, the bars show the 90% quantile and the length of the whiskers corresponds to the standard deviation of the error. The errors were estimated using the MAF >1% SNP set.

The advantage of the RAS correction was diminished when comparing the SNP-specific differences of the RAS values between the two groups (Figure 2). However, the k-correction method performed best among all methods giving a 90% quantile (median) error regarding the RAS value differences between both DNA pools of 0.056 (0.021) after filtering SNPs with a MAF ≤1%. The second best estimation of the SNP-specific differences between both pools was provided by the uncorrected RAS values obtained from the Birdseed2 calling algorithm with a 90% quantile (median) error of 0.056 (0.022). The corresponding values of the error distribution of these two methods using the complete SNP set were 0.062 (0.023) and 0.061 (0.023), respectively, whereas the differences based on the Birdseed2 RAS values were the most accurate among all methods tested. Analyzing the differences of the RAS values between both DNA-pools, all but the k-corrected methods were less accurate compared to the methods using the uncorrected values, regardless of the MAF filter applied. Filtering SNPs with a MAF ≤5% did not result in a better overall accuracy compared to the MAF ≤1% filtered SNPs (Table 2).

**Table 2:** Error of the RAS value differences between both DNA pools

	**RAS (Birdseed2)**		**RAS (CEL)**		**RAS (k-corrected)**		**RAS (PPC-corrected)**	
**SNP set**	**Q90 (Median)**	**Mean (SD)**	**Q90 (Median)**	**Mean (SD)**	**Q90 (Median)**	**Mean (SD)**	**Q90 (Median)**	**Mean (SD)**
all SNPs (N = 772729)	0.0613 (0.0229)	0.0287 (0.024)	0.0635 (0.0243)	0.03 (0.024)	0.0617 (0.0231)	0.0289 (0.024)	NA	NA
10000 SNPs MAF >1%	0.0562 (0.0218)	0.0266 (0.022)	0.0583 (0.0227)	0.0277 (0.022)	0.0556 (0.0213)	0.0262 (0.021)	0.0616 (0.0229)	0.0288 (0.024)
10000 SNPs MAF >5%	0.0578 (0.0229)	0.0275 (0.022)	0.0601 (0.0236)	0.0286 (0.022)	0.0574 (0.0226)	0.0272 (0.022)	NA	NA
	**RAS (tan-corrected n = 0.5)**		**RAS (tan-corrected n = 1)**		**RAS (tan-corrected n = 2)**			
**SNP set**	**Q90 (Median)**	**Mean (SD)**	**Q90 (Median)**	**Mean (SD)**	**Q90 (Median)**	**Mean (SD)**		
all SNPs (N = 772729)	0.0976 (0.0346)	0.0457 (0.11)	0.102 (0.0358)	0.0488 (0.19)	0.123 (0.0390)	0.106 (3.1)		
10000 SNPs MAF >1%	0.0671 (0.0266)	0.0322 (0.025)	0.0675 (0.0271)	0.0326 (0.026)	0.0715 (0.0285)	0.0345 (0.028)		
10000 SNPs MAF >5%	0.067 (0.0269)	0.0325 (0.025)	0.0667 (0.0270)	0.0325 (0.025)	0.068 (0.0274)	0.0331 (0.026)		

Absolute differences (error) of the RAS value differences between both DNA pools and the difference of the expected RAS values calculated from calls obtained by individual genotyping of the same DNA samples. Q90 represents the 90% quantile, SD the standard deviation of the error.

## Discussion

The pooling-specific error of the Affymetrix Genome-Wide Human SNP Array 6.0 in the context of allelotyping studies and the optimal method for correction strongly depend on the study design. When comparing allele frequencies determined using pooled DNA samples with those obtained by individual genotyping, an appropriate correction for RAS values may dramatically increase the overall accuracy. Uncorrected RAS values are, however, almost as reliable as (if not even better than) corrected values when comparing the differences in allele frequencies between two pooled DNA sample groups. This finding is of great relevance, since the correction algorithms require prior knowledge of correction values for each SNP, which are array specific.

In detail, the k-correction method requires intensity values of heterozygote individuals, the PPC and tan-correction require intensity values from all three genotypes of each SNP, which are difficult to obtain, especially for rare SNPs. For both the k-correction and the tan-correction algorithms, correction values are calculated only once for each SNP as the mean of the RAS values obtained from the individually genotyped samples. In contrast, for the PPC method the RAS values of each individually genotyped A and B probe pair have to be included in a regression model, and, in a second step, the median of all corrected probe-specific RAS values needs to be calculated per SNP. This results in a large computational effort and increased data handling. The PPC algorithm was originally developed using approximately 8000 SNPs of the former Affymetrix GeneChip 10 K Mapping array, which included 10 probes per SNP distributed over both target DNA strands. This probe-based correction was implemented to compensate for the specific hybridization efficiencies of the probe pairs [11]. Since the Affymetrix Genome-Wide Human SNP Array 6.0 was designed by selecting only 3–4 perfect match probe pairs per SNP that represent the most reliable [15], we implemented the PPC correction algorithm on a per-SNP base using the RAS values obtained from the Birdseed2 calling algorithm. The accuracy of the 10,000 SNPs tested using the adapted PPC correction method was comparable to the accuracy of using the Affymetrix GeneChip 10 K Mapping array (Table 1 and [11]), but markedly reduced computation time.

At least for comparing differences between allele frequencies of DNA pools, the use of uncorrected RAS values, obtained from the intensity values of the Birdseed2 calling algorithm, may represent a reliable and easy-to-implement approach.

There are several limitations of the present study including the lack of a more detailed examination of the SNP-specific pooling error and an analysis of the impact of different numbers of pool replicates. Since the number of individual DNA samples combined in the pools should not significantly affect the pooling-specific error [14], we decided to use 50 DNA samples per pool and two true replicates for each of the two DNA pools. This approach has been shown to be more effective with respect to the reduction of the pooling-specific error than just replicating the amplification to hybridization steps of the same DNA pool [11]. A limitation of both the PPC and the tan-correction method is that the RAS values may be out of the 0–1 range. This affected 4.4% (pool 1) and 4.7% (pool 2) of the SNPs with a MAF >1% for the tan-correction method, and proportions were similar for the PPC correction. These values were higher than the number of SNPs reported in [11], which might be due to the different array design and fewer numbers of technical replicates in our study.

## Conclusion

The comparison of the allele frequency differences between two DNA pools were more accurate than the comparison of the allele frequencies of a DNA pool with those obtained from individual genotyping. Removing SNPs with a MAF <1% notably increased the accuracy especially in the latter scenario. Compared to the MAF <1% filtered SNP set, additional removal of all SNPs with a MAF <5% resulted, if at all, in an only marginally increased accuracy.

Other studies already estimated the pooling-specific error using earlier Affymetrix genotyping array models [8-12]. Here we present the results of an estimation of the pooling-specific error on a genome-wide scale using pools of 50 individual DNA samples genotyped with the Affymetrix Genome-Wide Human SNP Array 6.0. We show that results comparable with those obtained using the earlier Affymetrix mapping array systems are achievable, despite the missing mismatch probes and fewer number of probe pairs per SNP. These results demonstrate the theoretical reliability of this array system for analyzing pooled DNA samples on a genome-wide scale as a cost saving alternative, especially when searching for moderate or strong genetic effects [5,6]. Nevertheless, due to the pooling-specific error, detection of small genetic effects may be hard, even for common SNP variants. If allelotyping is part of a multi-stage design combined with a screening stage by individual genotyping and a replication stage, the cost savings that can be achieved by the pooling approach depend on several factors like the number of pool replicates, the genetic effect sizes that are present and on the costs per genotype compared to individual genotyping. Depending on the combination of these factors it could, under certain circumstances, be more cost effective to use individual genotyping instead of the pooling approach. Details on the cost estimation are given in [16].

Based on the results of this study, we suggest the following approach for analyzing pooled data in the given scenarios. When comparing pooled DNA samples of one group with individual genotypes from another group, and if correction values of all three genotypes per SNP are available, we recommend to use the tan-correction with parameter *n* = 0.5. In case an association analysis using DNA pools in both groups is to be conducted, the k-correction seems to be a slightly more precise method (if corrections values of heterozygous SNP calls are available) than using uncorrected RAS values obtained from the Birdseed2 calling algorithm being the second best option. In any case, we suggest to focus on SNPs with a MAF >1%.

## Methods

Array data from 4096 individuals of the Study of Health in Pomerania (SHIP) [17], a population-based cohort in the northeast of Germany were used to determine allelotyping-specific SNP correction values. For estimating the pooling-specific error, 100 individuals of European ancestry (51 males and 49 females) were randomly selected from the same study. The study followed the recommendations of the Declaration of Helsinki. The study protocol was approved by the medical ethics committee of the University of Greifswald. Written informed consent was obtained from each of the study participants.

### Preparation and purification of DNA

Chromosomal DNA was purified from whole blood samples using the QIAamp DNA blood mini kit (Qiagen, Hilden, Germany) following the manufacturer’s instruction. To remove contaminating RNA, the optional RNase A treatment step (30 min at RT with 20 μl Ribonuclease A (20 mg/ml)) was routinely performed. The DNA quantity was measured with a NanoDrop® ND-1000 UV–vis Spectrophotometer (NanoDrop Technologies) and the integrity of the DNA preparations was analyzed by electrophoresis using 0.8% Agarose-1x TBE gels stained with ethidium bromide. Only DNA preparations showing no signs of degradation at all were used for allelotyping.

### Obtaining individual genotype calls

The 4096 DNA samples used to determine the correction values which included the 100 samples used for estimation of the pooling-specific error were individually genotyped using the Affymetrix Genome-Wide Human SNP Array 6.0. Processing of genomic DNA and array hybridization were performed in accordance with the manufacturer’s standard recommendations. Genotypes were determined using the Birdseed2 calling algorithm [13]. For quality control purposes, several control samples where added. On the chip level, only subjects with a genotyping rate on QC probe sets (QC call rate) of at least 86% were included. Finally, all arrays demonstrated a sample call rate >92% (average call rate of 99.75%). Altogether, 909,622 SNP genotypes were successfully typed.

Only SNPs with a call rate of 100% where used for estimating the pooling-specific error. Of these 772,731 SNPs, two subsets of 10,000 SNPs with MAFs >1% and >5%, respectively, were selected and used in corresponding subset analyses. All but 147 SNPs included only in the overall SNP set were in Hardy-Weinberg equilibrium (p_HWE_ > 0.001).

### Preparation and purification of equimolar DNA pools for the Allelotyping analysis

For the allelotyping analysis, two disjunct DNA pools (Pool 1 and Pool 2), each consisting of equimolar DNA samples from 50 individuals (24 and 25 female samples per pool, respectively), were prepared. As pilot experiments had revealed that it is extremely important that the individual DNA samples in the pools are indeed present in equimolar amounts, the pooling of the individual DNA samples was carried out with maximal pipetting accuracy. Subsequently, the pools were purified by an additional phenol-chloroform extraction procedure including a final ethanol precipitation, because the pilot studies had also demonstrated that this treatment strongly improves the results of the array analyses. This purification was performed as follows: The total volume of the respective DNA pool was adjusted to 200 μl using 1xTE buffer in a reaction tube. To this DNA solution, 200 μl TE-equilibrated (pH 7.5 – 8.0) Phenol solution (Roti-Phenol, Carl Roth, Karlsruhe, Germany) was added and the resulting mixture was manually mixed for 1 min. Subsequent phase separation by centrifugation for 15 minutes at room temperature and 8000 rpm was accomplished using a bench-top centrifuge. Upper and middle phase were completely transferred to a new reaction tube, and again 100 μl TE-equilibrated Phenol and a 100 μl Chloroform/isoamyl alcohol mix (24:1 ratio) were added. The resulting mixture was manually shaken for 1 min. The centrifugation step was repeated and upper and middle phase were again transferred to a new reaction tube. Next, 200 μl of chloroform/isoamyl alcohol mix (24:1) were added, and the resulting mixture was manually shaken for 1 min. The subsequent centrifugation step was carried out at room temperature for 5 min at 13000 rpm. The upper and middle phases were transferred to a new reaction tube, and the complete chloroform/isoamyl alcohol extraction step was repeated. Subsequently, only the upper phase was transferred to a new reaction tube, leaving the middle phase behind. After adding 40 μl of 3 M sodium acetate (pH 5.2) and thoroughly mixing, 1 ml of ethanol was added. The mixture was left overnight at – 20°C for precipitation of the sodium-DNA salts. The precipitate was harvested by centrifugation for 15 min at 4°C and 13000 rpm. After removing the supernatant, 1 ml of 75% ethanol was added and the DNA-sodium pellet was washed by repeatedly inverting the reaction tube to remove excess sodium-acetate salts. The preceding centrifugation step was repeated and the supernatant carefully removed. The pellet was dried in a vacuum centrifuge for 20 min, and resuspended in 30 μl 1x TE buffer for 1 h at 37°C under agitation using a thermomixer (Eppendorf) at 500 rpm. The DNA concentration was again determined using a NanoDrop Spectrophotometer, and the integrity of the DNA preparations was analyzed by electrophoresis using 0.8% Agarose-1xTBE gels stained with ethidium bromide.

Two technical replicates were performed with each DNA pool. To this purpose, both pools were independently processed two times, starting with enzymatic DNA restriction and ending up with array hybridization, detection, and scanning.

**Figure 3:**
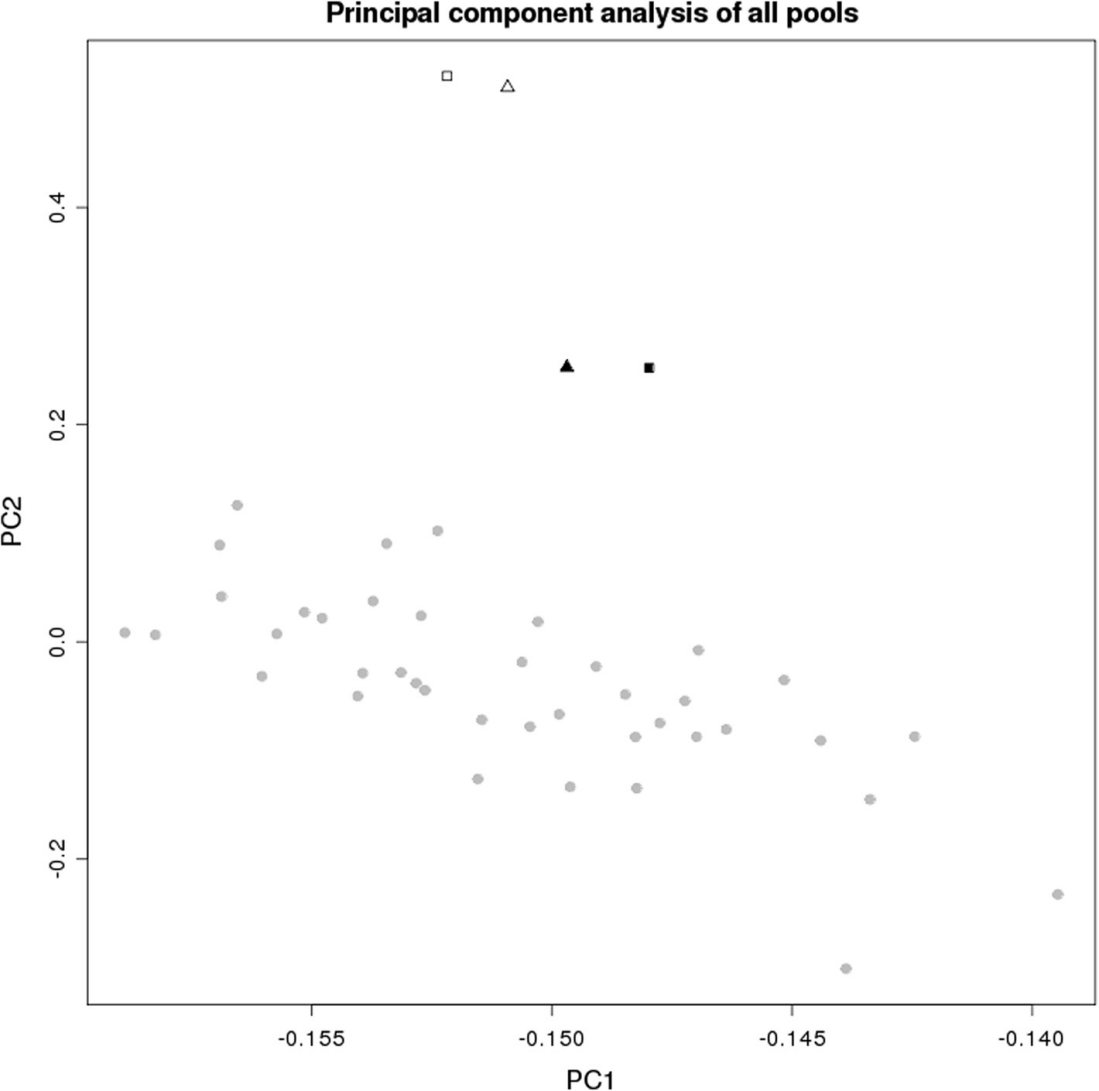
**PCA plot of all arrays.** The results of the principal component analysis of all 44 arrays were used as input for the Birdseed2-based intensity calculation. The x-axis represents the first and the y-axis the second principal component based on the RAS values of all 909,622 SNPs represented on the array. Triangles correspond to the arrays of DNA-Pool 1 and squares to the arrays of DNA-Pool 2. Equally colored symbols indicate that the corresponding DNA-pools were processed by the same person.

As quality control, a principal component analysis (PCA) was performed on all arrays that were used as input for the Birdseed2 calling algorithm for obtaining the RAS values. Arrays that were identified as outliers were discarded, and array analysis was repeated for these samples. Finally, for all arrays, both first two principal components were within the range of ±4 standard deviations of their mean. The PCA results are shown in Figure 3.

**Figure 4:**
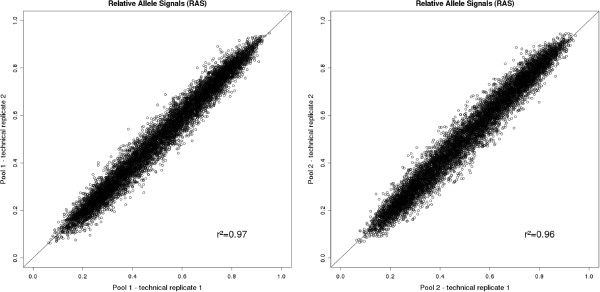
**Scatter plot of technical replicates.** RAS values are based on intensities obtained from the Birdseed2 calling algorithm using the MAF >1% SNP set between both technical replicates of Pool 1 (left plot) and Pool 2 (right plot), respectively.

The r^2^ value between two technical pool replicates based on the RAS values obtained by Birdseed2 of 10,000 SNPs with a MAF >1% was 0.97 and 0.96 for pool 1 and pool 2, respectively (Figure 4).

All arrays were scanned and the corresponding CEL files were generated using the Affymetrix® GeneChip® Genotyping Analysis Software Version 4.0 [18].

If not specified otherwise, RAS values of all technical replicates of a DNA pool were averaged on a per-SNP basis prior to further analysis. If corrected RAS values were used, correction was performed before this averaging step.

### Determination of the RAS values

For calls obtained from individual genotyping, the relative intensity values for allele A were set to 1, 0.5, and 0 for genotypes AA, AB, and BB, respectively, and averaged over all individuals of the corresponding pool to determine the respective RAS value.

For the DNA pools, the intensity values for the A and B alleles of each SNP were obtained from the CEL files using the Birdseed2 calling algorithm as implemented in the Affymetrix Power Tools (APT version 1.10.1). To accomplish the minimal number of genotyping arrays necessary to apply this algorithm, we included 40 additional DNA pools, also originating from individuals of European ancestry. The DNA pools were prepared in the course of a different allelotyping project (genetic determinants for breast cancer susceptibility) and were processed using the same protocol and array system (Affymetrix Genome-Wide Human SNP Array 6.0). The CEL file intensity values, the output from the Birdseed2 algorithm (CHP files) as well as the individual genotype calls were imported into a database build on Caché (InterSystems Corporation, Cambridge, MA, USA) for further analyses.

The RAS values based on the Birdseed2 intensity values were calculated for each SNP as RAS = SignalA/(SignalA + SignalB), where the SignalA- and SignalB-columns of the corresponding CHP files were used. For the CEL file intensity based RAS-values, we obtained the signal intensities for alleles A and B by calculating the median probe intensities from all probes of a SNP for alleles A and B, respectively, and used those values as input in the RAS computation formula.

### Correction of intensity values

Ideally, the RAS values for a given individually genotyped AB SNP will amount to 0.5 (heterozygous, AB), 1 (homozygous, AA) and 0 (homozygous, BB), respectively. In practice, the values obtained for each probe set deviate from this ideal due to differential hybridization efficiencies. Therefore, if RAS values generated using pooled DNA samples from one group (e. g. patients) shall be compared to averaged SNP-specific call values generated by individual genotyping of a different group (e. g. healthy control individuals), a correction step has to be included to compensate for this hybridization-specific bias [19]. One option is provided by the so called k-correction [8,12,14,20], which corrects the RAS value for heterozygote calls. The correction factor *c* is specific for each probe set and has to be determined empirically by individual array-based genotyping of heterozygous individuals. The k-corrected RAS value *K* for allele A of a given SNP was calculated as follows:

$$K=\mathrm{RAS}\left(1-c\right)/\left(\left(1-\mathrm{RAS}\right)*c+\mathrm{RAS}\left(1-c\right)\right),$$

with 0 ≤ RAS ≤ 1 and 0 < *c* < 1, where the correction value *c* is the mean RAS value of the individual heterozygous calls, RAS is the observed relative allele frequency and *K* is the k-corrected RAS value.

In contrast to the k-correction, the PPC correction algorithm uses the intensity values of all individually genotyped homozygous AA and BB as well as all heterozygous AB samples as input and fits for each SNP a second-degree polynomial using the relative intensity value of a probe as independent variable. Our calculation was done according to the R-script provided in [11] using the RAS values obtained from the Birdseed2 algorithm as independent variables (omitting the calculation of the median of the probe-specific corrected RAS values).

### Tangents based correction method

**Figure 5:**
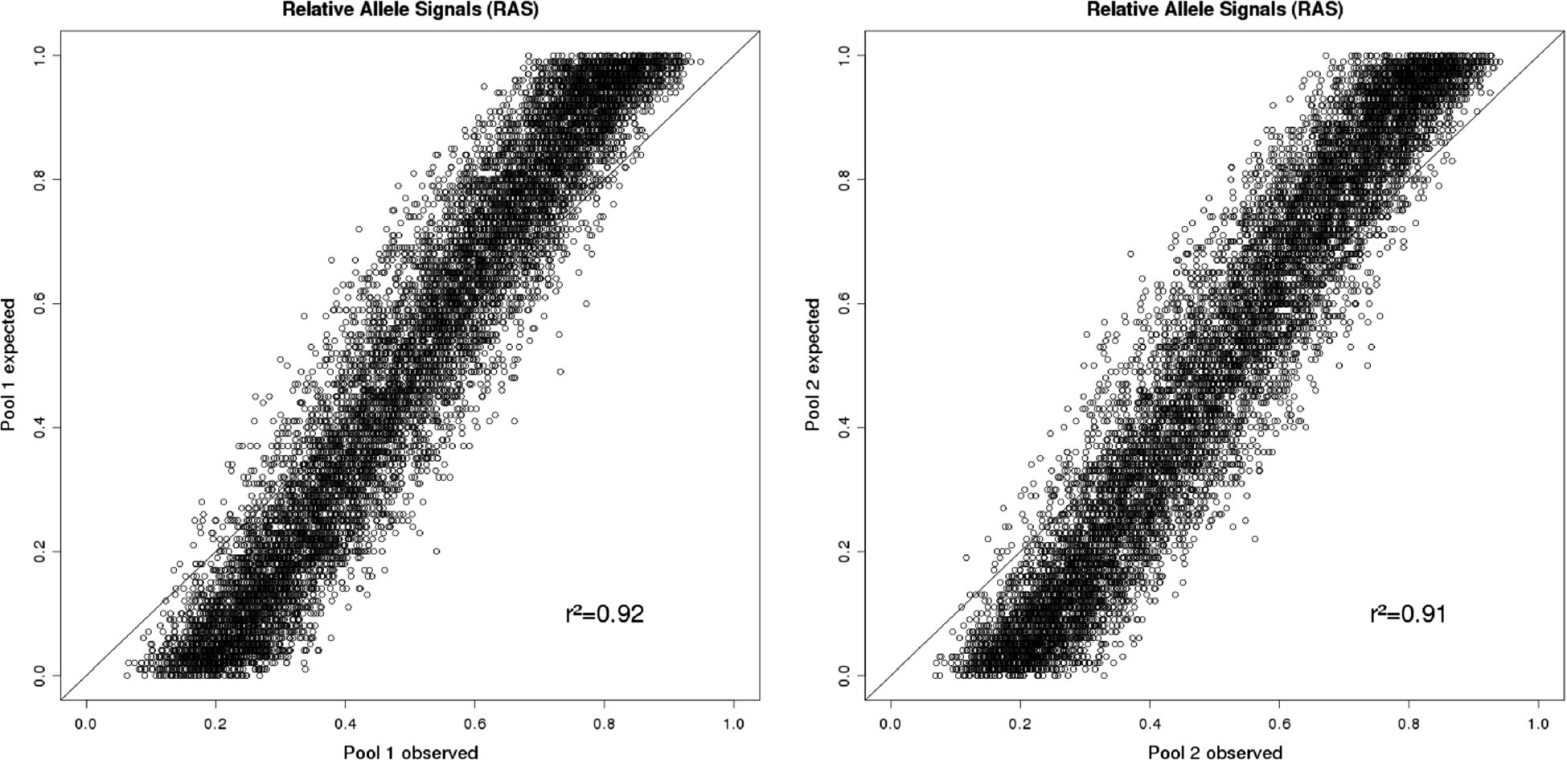
**Scatter plot of observed vs. expected RAS values.** Scatter plots showing the RAS values based on intensities obtained from pooled DNA samples using the Birdseed2 calling algorithm and the MAF >1% SNP set (x-axis) against the expected RAS values calculated from calls obtained by individual genotyping (y-axis) using the same DNA samples. The left plot corresponds to DNA-Pool 1 and the right to DNA-Pool 2.

When comparing uncorrected RAS values from pools with the corresponding allele frequencies obtained from individual genotyping, values close to 0 or 1 seem to deviate more from the expected frequencies than values closer to 0.5 (Figure 5). Therefore, the distribution of the RAS values and the magnitude of correction depend on the true allele frequencies, which we modeled by an arctangent function with a free parameter. For each SNP, three correction values were required as input: the mean RAS values of individuals with the genotypes BB, AB and AA, named c0, c1, c2, respectively. These correction values were obtained from the Birdseed2 intensity values of 4096 DNA samples individually genotyped using the Affymetrix Genome-Wide Human SNP Array 6.0. The mean and standard deviation of the correction values of all 909,622 SNPs represented on the array were c0 = 0.23 ± 0.04, c1 = 0.51 ± 0.08 and c2 = 0.78 ± 0.07.

**Figure 6:**
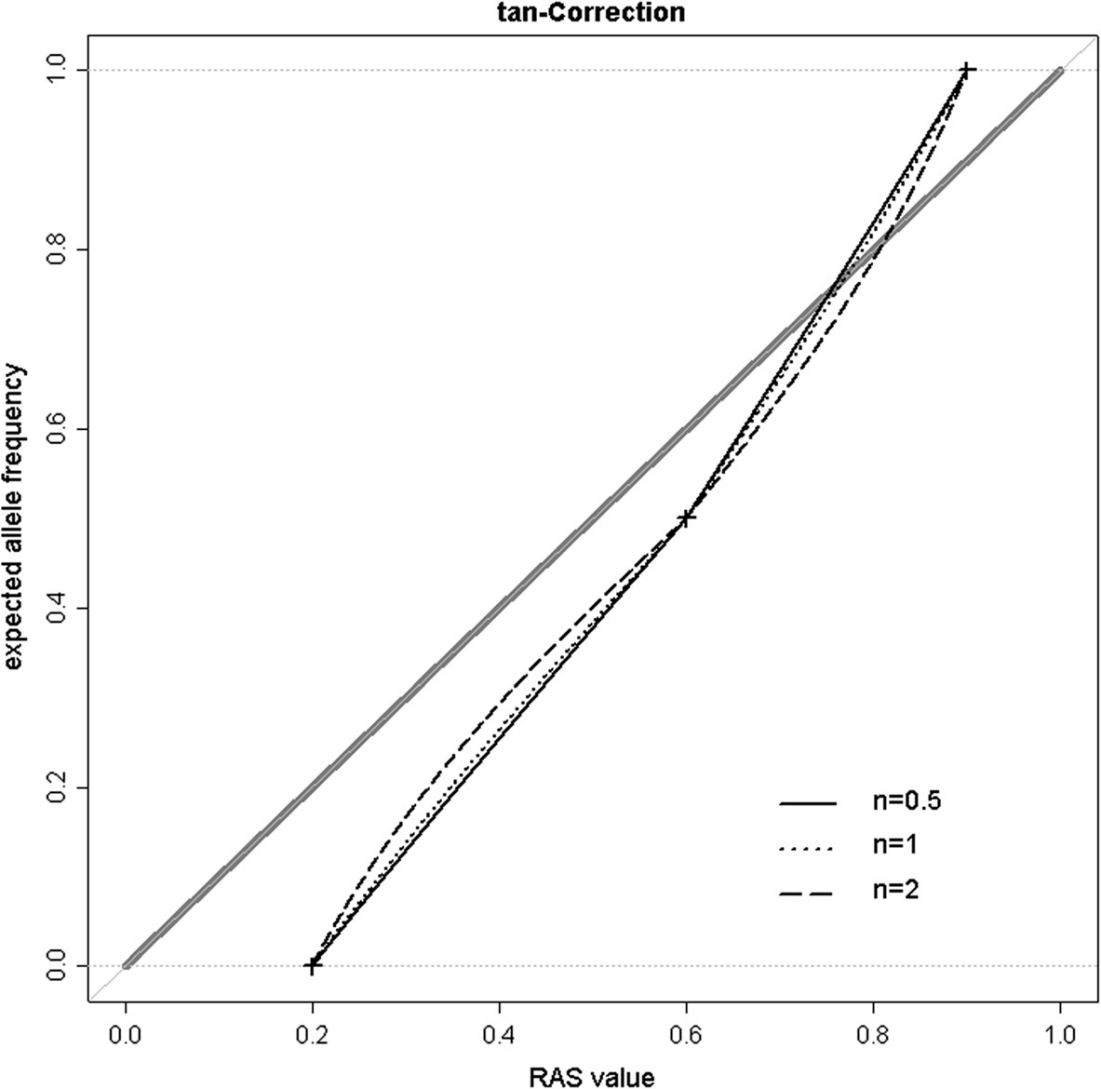
**An example for tan-correction.** Expected RAS value distribution (y-axis) depending on observed RAS values (x-axis) modeled using arctangent functions as described in the methods section. In this example, the mean RAS values (correction values) of the BB, AB and AA genotypes were set to 0.15, 0.55 and 0.8, respectively, and labeled by crosses. The gray diagonal corresponds to the tan-corrected RAS values. The black solid, dotted and dashed lines represent the model function by setting the parameter *n* to the values of 0.5, 1 and 2, respectively.

Two separate arctangent functions of the form

$${\mathrm{RAS}}_{\mathrm{obs}}=\arctan\left(n*x+m\right)*b+a$$

were used to model the observed RAS value of a SNP (RAS_obs_): one with parameters for observed RAS values below the correction value of the AB genotype (c1) and the other for RAS_obs_ values above this correction value (Figure 6). The parameters *a*, *b* and *m* were chosen in such a way that the RAS_obs_ value of the true A allele frequency *x* results in c0, c1 and c2 for *x* = 0, 0.5 and 1, respectively. The parameter *n* influences the convexity of the curve and was tested with the values of 0.5, 1 and 2. The correction of the RAS values was performed by solving the equation for *x*.

Therefore, the corrected RAS value RAS_corr_ could be calculated by the formula

$${\mathrm{RAS}}_{\mathrm{corr}}=\left(\tan\left(\left({\mathrm{RAS}}_{\mathrm{obs}}-a\right)/b\right)-m\right)/n,$$

with *m* = −*n*/2, *a* = c1 and *b* = (c0-c1)/arctan(*m*) for RAS_obs_ ≤ c1 and *b* = (c2-c1)/arctan(*n* + *m*) for RAS_obs_ ≥ c1.

If any SNP correction values were missing, e.g. due to lack of genotyped individuals of the respective genotype, the uncorrected RAS value was used. This was the case for 1.87% of the 10,000 SNPs of the 1% MAF-filtered SNP set.

The intensity values, RAS values and genotype calls were stored in a database build on Caché (InterSystems Corporation, Cambridge, MA, USA). Computations and data management were performed in Caché, InforSense (ID Business Solutions Ltd., Guildford, Surrey, UK), R [21] and the Perl 5 programming language (http://www.perl.org). A Perl script for calculating and testing allele-specific differences between pools using uncorrected RAS values, k-correction or tan-correction was developed and successfully implemented in the allelotyping workflow used in [6] and is available upon request.

The array data of each technical replicate of Pool 1 and Pool 2 as well as the correction values used for the k-correction and tan-correction are available at the GEO (Gene Expression Omnibus) public repository under accession number GSE48190. Additional file 1 and Additional file 2 contain the expected RAS values of Pool 1 and Pool 2, respectively. Additional file 3 contains the correction values calculated for the PPC correction of the 10,000 SNPs with MAF >1% and Additional file 4 lists all 10,000 SNPs included in the MAFs >5% subset.

## Competing interests

The authors declare that they have no competing interests.

## Authors’ contributions

AT performed all *in silico* analyses and drafted the manuscript. AT, UV and GH designed the study. FDE, AW, KU, and AP performed isolation of DNA samples, preparation of DNA pools, and subsequent sample processing including array hybridization. MN and HV participated in design and coordination of the SHIP study. FDE, UV, HV and GH revised the manuscript. All authors read and approved the final manuscript.

## Supplementary Material

Additional file 1 **Expected RAS values of Pool 1.** Table of expected RAS values calculated from calls (MEANcallPool) and average RAS values using the Birdseed2 calling algorithm (MEANindRASPool) obtained by individual genotyping of all DNA samples included in Pool 1. The column numElementsPool denotes the number of successfully genotyped samples per SNP (probeset). Click here for file

Additional file 2 **Expected RAS values of Pool 2.** Table of expected RAS values calculated from calls (MEANcallPool) and average RAS values using the Birdseed2 calling algorithm (MEANindRASPool) obtained by individual genotyping of all DNA samples included in Pool 2. The column numElementsPool denotes the number of successfully genotyped samples per SNP (probeset). Click here for file

Additional file 3 **PPC correction values.** Table of the correction values used for the PPC correction of the subset of 10,000 SNPs with MAFs > 1%. For each SNP (Probeset) the columns b0, b1, b2 and Rsq denote the intercept, the effect of the RAS value, the effect of the squared RAS value and the R^2^ value of the PPC regression model, respectively, whereas n number of samples were included in the regression. Click here for file

Additional file 4 List of 10,000 SNPs included in the MAFs >5% subset. Click here for file

## References

[B1] Sham P Bader JS Craig I O’Donovan M Owen M DNA pooling: a tool for large-scale association studies Nat Rev Genet 2002 3 11 862 871 10.1038/nrg930 12415316

[B2] Liu Q Drgon T Walther D Johnson C Poleskaya O Hess J Uhl G Pooled association genome scanning: validation and use to identify addiction vulnerability loci in two samples Proc Natl Acad Sci USA 2005 102 33 11864 11869 10.1073/pnas.0500329102 16091475 PMC1183486

[B3] Papassotiropoulos A Stephan D Huentelman M Hoerndli F Craig D Pearson J Huynh K Brunner F Corneveaux J Osborne D Wollmer M Aerni A Coluccia D Hanggi J Mondadori C Buchmann A Reiman E Caselli R Henke K De Quervain D Common Kibra alleles are associated with human memory performance Science 2006 314 5798 475 478 10.1126/science.1129837 17053149

[B4] Pearson JV Huentelman MJ Halperin RF Tembe WD Melquist S Homer N Brun M Szelinger S Coon KD Zismann VL Webster JA Beach T Sando SB Aasly JO Heun R Jessen F Kolsch H Tsolaki M Daniilidou M Reiman EM Papassotiropoulos A Hutton ML Stephan DA Craig DW Identification of the genetic basis for complex disorders by use of pooling-based genomewide single-nucleotide-polymorphism association studies Am J Hum Genet 2007 80 126 139 10.1086/510686 17160900 PMC1785308

[B5] Chiang CWK Gajdos ZKZ Korn JM Kuruvilla FG Butler JL Hackett R Guiducci C Nguyen TT Wilks R Forrester T Haiman CA Henderson KD Marchand LL Henderson BE Palmert MR McKenzie CA Lyon HN Cooper RS Zhu X Hirschhorn JN Rapid assessment of genetic ancestry in populations of unknown origin by genome-wide genotyping of pooled samples PLoS Genet 2010 6 3 e1000866 10.1371/journal.pgen.1000866 20221249 PMC2832667

[B6] Gaj P Maryan N Hennig EE Ledwon JK Paziewska A Majewska A Karczmarski J Nesteruk M Wolski J Antoniewicz AA Przytulski K Rutkowski A Teumer A Homuth G Starzyńska T Regula J Ostrowski J Pooled sample-based GWAS: a cost-effective alternative for identifying colorectal and prostate cancer risk variants in the polish population PLoS One 2012 7 4 e35307 10.1371/journal.pone.0035307 22532847 PMC3331859

[B7] Barratt BJ Payne F Rance HE Nutland S Todd JA Clayton DG Identification of the sources of error in allele frequency estimations from pooled DNA indicates an optimal experimental design Ann Hum Genet 2002 66 5–6 393 405 10.1017/S0003480002001252 12485472

[B8] Butcher LM Meaburn E Liu L Fernandes C Hill L Al-Chalabi A Plomin R Schalkwyk L Craig IW Genotyping pooled DNA on microarrays: a systematic genome screen of thousands of SNPs in large samples to detect QTLs for complex traits Behav Genet 2004 34 5 549 555 10.1023/B:BEGE.0000038493.26202.d3 15319578

[B9] Butcher LM Meaburn E Knight J Sham PC Schalkwyk LC Craig IW Plomin R SNPs, microarrays and pooled DNA: identification of four loci associated with mild mental impairment in a sample of 6000 children Hum Mol Genet 2005 14 10 1315 1325 10.1093/hmg/ddi142 15800012

[B10] Meaburn E Butcher LM Liu L Fernandes C Hansen V Al-Chalabi A Plomin R Craig I Schalkwyk LC Genotyping DNA pools on microarrays: tackling the QTL problem of large samples and large numbers of SNPs BMC Genomics 2005 6 52 10.1186/1471-2164-6-52 15811185 PMC1079828

[B11] Brohede J Dunne R McKay JD Hannan GN PPC: an algorithm for accurate estimation of SNP allele frequencies in small equimolar pools of DNA using data from high density microarrays Nucleic Acids Res 2005 33 17 e142 10.1093/nar/gni142 16199750 PMC1240117

[B12] Meaburn E Butcher L Schalkwyk L Plomin R Genotyping pooled DNA using 100K SNP microarrays: a step towards genomewide association scans Nucleic Acids Res 2006 34 4 e27 10.1093/nar/gnj027 16478714 PMC1368655

[B13] Korn JM Kuruvilla FG McCarroll SA Wysoker A Nemesh J Cawley S Hubbell E Veitch J Collins PJ Darvishi K Lee C Nizzari MM Gabriel SB Purcell S Daly MJ Altshuler D Integrated genotype calling and association analysis of SNPs, common copy number polymorphisms and rare CNVs Nat Genet 2008 40 10 1253 1260 10.1038/ng.237 18776909 PMC2756534

[B14] Le Hellard S Ballereau S Visscher P Torrance H Pinson J Morris S Thomson M Semple C Muir W Blackwood D Porteous D Evans K SNP genotyping on pooled DNAs: comparison of genotyping technologies and a semi automated method for data storage and analysis Nucleic Acids Res 2002 30 15 e74 http://nar.oxfordjournals.org/content/30/15/e74.long 10.1093/nar/gnf070 12140336 PMC137092

[B15] McCarroll SA Kuruvilla FG Korn JM Cawley S Nemesh J Wysoker A Shapero MH de Bakker PIW Maller JB Kirby A Elliott AL Parkin M Hubbell E Webster T Mei R Veitch J Collins PJ Handsaker R Lincoln S Nizzari M Blume J Jones KW Rava R Daly MJ Gabriel SB Altshuler D Integrated detection and population-genetic analysis of SNPs and copy number variation Nat Genet 2008 40 10 1166 1174 10.1038/ng.238 18776908

[B16] Chiang CWK Gajdos ZKZ Korn JM Butler JL Hackett R Guiducci C Nguyen TT Wilks R Forrester T Henderson KD Marchand LL Henderson BE Haiman CA Cooper RS Lyon HN Zhu X McKenzie CA Palmert MR Hirschhorn JN The efficacy of detecting variants with small effects on the Affymetrix 6.0 platform using pooled DNA Hum Genet 2011 130 5 607 621 10.1007/s00439-011-0974-0 21424828 PMC3474315

[B17] Völzke H Alte D Schmidt CO Radke D Lorbeer R Friedrich N Aumann N Lau K Piontek M Born G Havemann C Ittermann T Schipf S Haring R Baumeister SE Wallaschofski H Nauck M Frick S Arnold A Jünger M Mayerle J Kraft M Lerch MM Dörr M Reffelmann T Empen K Felix SB Obst A Koch B Gläser S Ewert R Fietze I Penzel T Dören M Rathmann W Haerting J Hannemann M Röpcke J Schminke U Jürgens C Tost F Rettig R Kors JA Ungerer S Hegenscheid K Kühn JP Kühn J Hosten N Puls R Henke J Gloger O Teumer A Homuth G Völker U Schwahn C Holtfreter B Polzer I Kohlmann T Grabe HJ Rosskopf D Kroemer HK Kocher T Biffar R John U Hoffmann W Cohort profile: the study of health in Pomerania Int J Epidemiol 2011 40 2 294 307 10.1093/ije/dyp394 20167617

[B18] Affymetrix, Inc Affymetrix® GeneChip® genotyping analysis software User’s guide version 4.0 2005 Affymetrix, Inc http://www.affymetrix.com/Auth/support/downloads/manuals/gtype_user_guide.pdf

[B19] Simpson C Knight J Butcher L Hansen V Meaburn E Schalkwyk L Craig I Powell J Sham P Al-Chalabi A A central resource for accurate allele frequency estimation from pooled DNA genotyped on DNA microarrays Nucleic Acids Res 2005 33 3 e25 10.1093/nar/gni028 15701753 PMC549427

[B20] Craig D Huentelman M Hu-Lince D Zismann V Kruer M Lee A Puffenberger E Pearson J Stephan D Identification of disease causing loci using an array-based genotyping approach on pooled DNA BMC Genomics 2005 6 138 10.1186/1471-2164-6-138 16197552 PMC1262713

[B21] R Development Core Team R: A Language and Environment for Statistical Computing 2006 Vienna, Austria: R Foundation for Statistical Computing http://www.R-project.org

